# Profile of *DHX37* gene defects in human genetic diseases: 46,XY disorders of sex development

**DOI:** 10.3389/fendo.2025.1507749

**Published:** 2025-02-14

**Authors:** Huifang Peng, Wenyuan Peng, Jiali Chen, Keyan Hu, Yingyu Zhang, Yujin Ma, Hongwei Jiang

**Affiliations:** Henan Key Laboratory of Rare Diseases, The First Affiliated Hospital, and College of Clinical Medicine of Henan University of Science and Technology, Luoyang, China

**Keywords:** *DHX37*, 46,XY DSD, RNA helicase, human genetic diseases, genetic defects

## Abstract

The RNA helicase *DHX37* gene is involved in ribosomal biological processes, and linked to human genetic diseases associated with 46,XY disorders of sex development (46,XY DSD) or neurodevelopment. Recently, relevant reports have primarily focused on 46,XY DSD. However, there is still a lack of overall understanding of the genetic characteristics, phenotype, etc. of the *DHX37* gene in human genetic diseases, and its molecular mechanism is not fully understood. We searched literature databases and summarized and analyzed all the literature related to *DHX37* to date, including case reports, cohort studies, and molecular mechanism studies, to comprehensively demonstrate the role of *DHX37* in human genetic diseases. Sixty patients were reported to have *DHX37*-related 46,XY DSD, with p.R308Q, p.R674W variants being the two most common mutation hotspots, accounting for 36.67% and 11.67% of cases respectively. In DSD cohorts, *DHX37* gene mutations have different detection frequencies (0.77%–45.45%), whereas in testicular regression syndrome and 46,XY gonadal dysgenesis cohorts, they have a high detection rate. The gonadal development and fertility of female (46,XX) carriers with *DHX37* gene mutations are not affected; however, incomplete penetrance may be observed in males (46,XY). The treatments are primarily surgical intervention and hormone replacement therapy administered at appropriate times; however, the long-term prognosis remains unknown. Although the molecular mechanism of *DHX37* mutation related 46,XY DSD is unclear, ribosome synthesis, cell cycle regulation, and the NF-κB and Wnt pathways may be affected. This review summarizes the profile of *DHX37* defects in human genetic diseases.

## 1 Overview of *DHX37* gene mutations in genetic diseases

**Figure 1:**
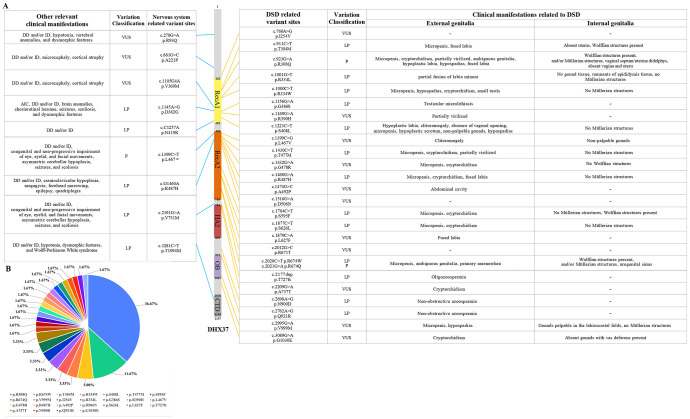
*DHX37* gene variants associated with 46,XY differential sex development (46,XY DSD) and the nervous system, along with their clinical manifestations. **(A)** Correspondence diagram showing DHX37 mutation sites, protein location, and clinical phenotypes. The left column describes other relevant clinical manifestations in patients with these variants, such as developmental delay, intellectual disability, hypotonia, and spinal abnormalities. The relevant specific genetic variants are listed in detail in the middle column. The specific locations of these variants and their functional domains are shown on the *DHX37* gene diagram in middle. The right column outlines the variant sites associated with DSD and their clinical manifestations, including abnormalities of the external and internal genitalia, such as micropenis, ambiguous genitalia, and absence of the uterus. It also indicates the clinical significance of the variant (LP or P). **(B)** The frequency of *DHX37* gene mutation sites. AIC, Aicardi syndrome, the related case involved was clinically diagnosed with AIC subsequently tested for DHX37 gene mutation, and we have retained this information. DD, developmental delay. ID, intellectual disability. LP, likely pathogenic. P, pathogenic. The DHX37 domain information data is sourced from the InterPro (https://www.ebi.ac.uk/interpro/protein/UniProt/Q8IY37/) and UniProt (https://www.uniprot.org/uniprotkb/Q8IY37/entry) databases.

DHX37 (NM_032656.4) belongs to the DexD/H-box RNA helicase family, a conserved protein group with an Asp-Glu-Ala-Asp motif (DEAD) (1). *DHX37* is associated with 46,XY disorders of sex development (46,XY DSD; 46,XY sex reversal 11 in OMIM #273250) as autosomal dominant inheritance, and developmental delay (“neurodevelopmental disorder with brain anomalies, with or without vertebral or cardiac anomalies” in OMIM #618731) as autosomal recessive inheritance (2, 3). We searched PubMed, Embase, and other literature databases and referred to the ClinVar gene variation database to summarize and display the *DHX37* gene variations and related clinical phenotype diagrams in **Figure 1**. There were 11 cohort studies and 6 case reports on *DHX37*-related 46,XY DSD, along with a little articles on neurodevelopmental disorders. We observed that the reported pathogenic or likely pathogenic mutations were mainly missense mutations, with only one frameshift mutation mentioned (**Figure 1A**). This characteristic is consistent across the phenotypes of 46,XY DSD or neurological system related phenotypes. Furthermore, another substantial feature of these variants is that mutations associated with both 46,XY DSD and the neurological system are highly concentrated in the two primary functional domains of *DHX37*, RecA1 (262-429 amino acid) and RecA2 (459-716 amino acid. The data is sourced from the InterPro and UniProt databases, website: https://www.ebi.ac.uk/interpro/protein/UniProt/Q8IY37/ and https://www.uniprot.org/uniprotkb/Q8IY37/entry). Although a greater number of variants have been reported to be associated with 46, XY DSD, there is no significant difference in the distribution of variants related to neurodevelopmental disorder in the domain structure.

This study mainly focuses on the situation of DHX37 in 46,XY DSD related fields. Of the 25 mutation sites associated with 46,XY DSD, 16 were located within these two domains. The RecA2 region has a higher frequency of mutations. Regarding the *DHX37*-related 46,XY DSD external genital phenotypes, micropenis was present in the majority of variant phenotypes, commonly accompanied by cryptorchidism. Both symptoms are widely present in mutations located within the RecA1 and RecA2 regions. Mutations in the RecA1 region display a broader range of external genitalia phenotypes, including clitoromegaly, absent vaginal opening, and hypospadias, which encompass more “female” phenotypes. Mutations in RecA2 predominantly manifest as micropenis and cryptorchidism. This characteristic combination is commonly observed in mutations affecting both the RecA1 and RecA2 regions of the *DHX37* gene. The co-occurrence of these two phenotypic features suggests a possible shared underlying mechanism in the developmental pathways influenced by mutations in these regions, potentially affecting penile growth and testicular descent. Regarding the internal genitalia, most mutations clustered in these two regions showed the absence of Müllerian structures, whereas Wolffian structures were present in some cases.

Sixty patients were reported to carry *DHX37* variants related to 46,XY DSD, with a total of 25 variant sites. Among them, the p.R308Q variant had the highest frequency, accounting for 36.67% (22/60); the p.R674W variant accounted for 11.67% (7/60); p.T304W variant;accounted for 5% (3/60); p.R334W, p.S408L, p.T477M, p.S595F, p.R674Q and p.V999M variants accounted for 3.33% (2/60) each; and the other 16 mutations, p.I254V, p.R334L, p.G386S, p.R390H, p.L467V, p.G478R, p.R487H, p.A492P, p.D506N, p.S626L, p.L627F, p.T727fs, p.A737T, p.N900D, p.Q921R and p.G1030E, accounted for 1.67% (1/60) each (**Figure 1B**). The two most common variants, p.R308Q and p.R674W, classified by the American College of Medical Genetics and Genomics classification as pathogenic (P) or likely pathogenic (LP), were located in the RecA1 and RecA2 regions, respectively. This aligns with our previous assertion that mutations within the RecA1 region tend to manifest more severe and diverse phenotypes. This distribution pattern highlights the potential functional importance of these specific amino acid positions within the DHX37 protein and their impact on sexual development. The clinical manifestations of the p.R308Q mutation locus include micropenis, cryptorchidism, hypoplasia of testicular tissue, testicular fibrosis, blurring of genitalia, and other clinical manifestations, such as clitoral hypertrophy and labial fusion in female patients without other systemic changes.

## 2 Variations of *DHX37* gene in 46,XY DSD cohorts

The first confirmation of an association between *DHX37* gene defect and 46,XY DSD was found via genetic screening of cohorts in 2019. In the cohort of 87 patients diagnosed with 46,XY DSD, including 55 patients with 46,XY GD and 32 patients with 46,XY DSD, after ruling out *LHCGR*, *AR*, *CYP17A1*, *HSD17B3*, *HSD3B2*, and *SRD5A2* gene defects, there were 17 patients with the “P” or “LP” mutation of *DHX37* gene, with a mutation frequency of 19.54% (17/87). Among the 14 patients with embryonic regression testicular syndrome (ETRS) in this cohort, seven had *DHX37* mutations, suggesting that DHX37 may be the main molecular cause of ETRS (50%, 7/14) (2). In another 46,XY DSD cohort of 145 patients, 13 (13/145, 8.97%) had a *DHX37* mutation. Of the 81 patients with 46,XY GD, nine carried a *DHX37* mutation (9/81, 11%), whereas in 16 patients with 46,XY testicular regression syndrome (TRS), four carried a *DHX37* mutation (4/16, 25%) (4). The above two cohort studies established that *DHX37* mutation is one reason of 46,XY DSD, and is more common in TRS. In a small cohort of patients with TRS and partial gonadal dysplasia (PGD) in Japan (n=11), five patients (5/11, 45.45%) had DHX37 mutations based on NGS-panel detection (5). In five ETRS patients from China, two of them (40%, 2/5) were detected p.R308Q of DHX37 at two months old and one year and 5 months old (6). In a 46,XY PGD cohort (n=25) from the United Kingdom and Chile, the *DHX37* mutation accounted for 16% (4/25), ranking second only to *NR5A1* gene mutation frequency (20%) (7). Using whole-exome sequencing (WES) in 140 patients with 46,XY DSD, *DHX37* mutations were detected in seven (7/140, 5%), with clinical phenotypes involving TRS (five patients), complete gonadal dysplasia (CGD) (one patient), and 46,XY DSD (one patient) (8). This study expanded the phenotype of 46,XY DSD caused by *DHX37* gene mutation by adding CGD. In a subsequent cohort of 46,XY CGD, a 31-year-old “female” with primary amenorrhea from the 46,XY “female” cohort (n=25) presented with ambiguous genitalia, Tanner stage as B1P3A2, Müllerian absence, ovaries absence, and a follicle stimulating hormone (FSH) level of 86.56 mIU/mL (normal range 2.5–10 mIU/mL). This patient had a 46,XY chromosome karyotype and *DHX37* c.1877C>T, p.S626L mutation (9). In a Brazilian cohort of 209 cases of 46,XY DSD, four patients carried *DHX37* “P” or “LP” mutations, with a frequency of 1.91% (4/209), identified using Sanger sequencing or massively parallel sequencing. Three additional patients were found to carry VUSs in *DHX37*, including two with mutations in other genes, which cannot be confirmed as a monogenic genetic cause. The detection frequency of *DHX37* mutation ranked sixth in this cohort (10). In a study of 54 Chinese 46,XY DSD cohorts, four patients carried the *DHX37* pathogenic variants detected using WES, with the fourth highest detection frequency of 7.41% (4/54). The top three detected genes were *AR*, *SRD5A2*, and *NR5A1* (11). There have also been reports of *DHX37* mutations in a posterior hypospadias cohort (2.38%, 1/42) (12). In a DSD cohort from Ukraine, WES was performed on 79 cases of 46,XY DSD, and the *DHX37* p.R674Q variant was detected in a three-year-old female patient (1/79, 1.27%) with an inguinal hernia and bilateral vesicoureteral reflux (13). In some patients, excised gonadal tissues were not pathologically examined before molecular diagnostic confirmation, and the extent of gonadal tissue differentiation and development is unknown. Notably, patients with the p.R308Q variant in some cohorts are almost exclusively females, who often present with primary amenorrhea, no uterine or ovarian tissue, enlarged clitoris, underdeveloped labia, and, in some cases, a vagina. These cohorts included female patients with 46,XY DSD of unknown etiology (4) and excluded patients with 17ß-hydroxysteroid dehydrogenase 3 deficiency, 5α-reductase type 2 deficiency, androgen insensitivity syndrome (7). In a cohort with 521 primary spermatogenic failure (SPGF) patient, 64 (12.28%, 64/521) men were molecular diagnosed finding in 39 genes, using clinical exomes sequencing. There were 4 patients carried DHX37 mutations as p.G386S, p.T727Dfs*60, p.N900D, p.Q921R (14). Overall, 12 studies of the 46,XY DSD cohort that mentioned different *DHX37* variations are shown in **Table 1**. The overall detection rate of 46,XY DSD was 12.280%–81.82%, whereas the detection rate of *DHX37* mutations was 0.77–45.45% in the differential cohort, which had various inclusion and exclusion criteria and patient numbers. Patients whose testes were not present in the scrotum or inguinal region on imaging or laparoscopic exploration, whose abdominal exploration showed testicular atrophy or gonadal dysgenesis, and whose anti-Müllerian hormone levels were below normal were included. This suggests that, in the presence of the above conditions, along with laboratory and imaging tests, genetic testing is necessary to determine whether a patient has gonadal dysgenesis. Variants in the *DHX37* gene were found in TRS and 46,XY GD cohorts, with a high rate of detection. For 46,XY GD, various studies have confirmed that variants in *NR5A1*, *SRY*, and *MAP3K1* are the most common causes of non-syndromic GD, with *DHX37* mutations also showing a relatively high detection frequency. For TRS or ETRS, *DHX37* mutation is the main genetic molecular etiology, accounting for 25%–50%; however, the sample sizes of the relevant TRS/ETRS cohorts were relatively small, necessitating large-scale screening.

**Table 1:** The *DHX37* gene in the 46,XY DSD cohorts.

No.	Time	Inclusion criteria	Molecular diagnostic rate	*DHX37* mutation frequency	Variant of *DHX37*	ACMG	Sex of rearing	Clinical manifestations	Reference
1	2019	Patients with 46,XY DSD without previous molecular diagnosis	–	19.54% (17/87)	c.G923A, p.R308Q	P	Male	Micropenis, small bilateral dysgenetic gonads, rudimentary fallopian tubes present	(2)
							Male	Micropenis, left gonad not found, right dysgenetic gonad,	
							Male	Micropenis, no gonadal tissue, rudimentary fallopian tubes present	
							Female	Micropenis, left gonad not found, small right dysgenetic gonad,	
							Male	Micropenis, no gonadal tissue	
							Male to female	Micropenis, no gonadal tissue	
							Female	Female, bilateral dysgenetic gonads	
					c.C2020T, p.R674W	LP	Male	Micropenis, small bilateral dysgenetic gonads	
							Male	Micropenis, small bilateral dysgenetic gonads	
							Male	Micropenis, no gonadal tissue	
							Male	Micropenis, no gonadal tissue	
							Male	Micropenis, right gonad not found, left dysgenetic testis with germ cell neoplasia in-situ	
							Male to female	Micropenis, no gonadal tissue	
							Female	Atypical external genitalia, bilateral dysgenetic gonads	
					c.C1784T, p.S595F	LP	Female	Atypical external genitalia, Previous gonadectomy, bilateral dysgenetic gonads	
							Male	Micropenis, no gonadal tissue	
					c.C911T, p.T304M	LP	Female	Previous genitoplasty	
2	2019	46,XY DSD of unknown etiology	–	8.97% (13/145)	c.G923A, p.R308Q	P	Female	Female external genitalia, primary amenorrhea, no Müllerian structures and Wolffian structures present, gonadal histology: R, small nodule of fibrous tissue; L, fibrous tissue with rare tubule-like structures	(4)
							Female	Poorly developed labia, absent uterus and vagina	
							Female	Virilized female, ambiguous genitalia, vagina present, absent uterus, Wolffian structures present	
							Female	Ambiguous genitalia, vaginal septum, and uterus didelphys	
							Male	Severe micropenis, cryptorchidism	
					c.G2021A, p.R674Q	P	Female	Female external genitalia, primary amenorrhea, no Müllerian structures and Wolffian structures present, gonadal histology: homogeneous fibrous tissues associated with a rete testis on both sides	
							Female	Female external genitalia, urogenital sinus, gonadal position: abdominal cavity, gonadal histology: bilateral fibrous gonads, duct-like structures, fragments of uterine tube	
					c.C911T, p.T304M	LP	Female	Female external genitalia, discrete fusion of labia minora, fallopian tube-like structures and epididymis on each side, no gonadal tissue	
							Female	Female external genitalia, absence of puberty, discrete fusion of labia minora, vagina present, absent uterus, Wolffian structures present (both sides), gonadal histology: homogeneous fibrous tissue	
					c.G1001T, p.R334L	LP	Female	Female external genitalia, partial fusion of labia minora, Vagina 16–17 mm long and 6–7 mm wide not opened, absent uterus; no gonads present, gonadal histology: L, no gonadal tissue, remnants of ductus deferens; R, no gonadal tissue, remnants of epididymis tissue	
							Male	Micropenis, hypospadias; unilateral cryptorchidism (L); small palpable testis (R), vagina present, gonadal histology: not available	
					c.C1877T, p.S626L	LP	Male	Micropenis and bilateral cryptorchidism, no Müllerian structures, gonadal position: L, Inguinal canal; R: abdominal cavity, gonadal histology: no gonadal tissue; remnants of epididymis, ductus deferens	
					c.G3089A, p.G1030E	VUS	Male	Bilateral cryptorchidism, absent gonads with vas deferens present (12 years), gonadal histology: not available	
3	2019^a^	46,XY women with unknown molecular etiology	30.8%	7.69% (4/52)	c.G923A, p.R308Q	P	Female	Partially virilized	(7)
							Female	Partially virilized	
							Female	Partially virilized, Müllerian structures: vaginal septum/uterine didelphys	
							Female	Partially virilized	
4	2021	46,XY DSD of unknown molecular etiology	–	5% (7/140)	c.G923A, p.R308Q	P	Male	Micropenis (<5 mm), posterior hypospadias, bilateral cryptorchidism, non-palpable gonads, hypoplastic labia, Müllerian ducts present	(8)
							Male	Micropenis (5 mm), bilateral cryptorchidism, non-palpable gonads, fused pigmented labia, fused labia minora	
							Male	Micropenis (12×9 mm), non-palpable gonads, bilateral cryptorchidism	
					c.C1000T, p.R334W	LP	Male	Micropenis (8×5 mm), midshaft hypospadias, non-palpable gonads, bilateral cryptorchidism	
					c.G1460A, p.R487H	LP	Male	Micropenis (<5 mm), bilateral cryptorchidism, non-palpable gonads, poorly developed and fused labia, gonadal position: abdominal cavity	
					c.G1169A, p.R390H	VUS	Female	External genitalia (as in females)	
					c.C1430T, p.T477M	LP	Male	Micropenis (15×6 mm), non-palpable gonads, bilateral cryptorchidism	
5	2022^b^	Nonsyndromic DSD with 46,XY	59.30%	6.22% (13/209)	c.C911T, p.T304M	LP	Female	DSDUE	(10)
					c.G923A, p.R308Q	P	Male	GD (ERTS)	
							Male to female	GD (ERTS)	
							Female	GD (partial)	
							Male	GD (ERTS)	
							Male	GD (ERTS)	
					c.C2020T, p.R674W	LP	Male to Female	GD (ERTS)	
							Female	GD (partial)	
					c.C1784T, p.S595F	LP	Female	GD (partial)	
							Male	GD (partial)	
					c.G2209A, p.A737T	VUS	Male	DSDUE	
					c.G1474C, p.A494P	VUS	Male	DSDUE	
					c.C1399G, p.L467V	VUS	Male	DSDUE	
6	2022^c^	Ambiguous external genitalia, delayed or incomplete puberty, virilization with typical female external genitalia, primary amenorrhea, breast development in a typical male, a discordance between the genital appearance, karyotype, and family history of DSD	43.00%	1.27% (1/79)	c.C2020T, p.R674W	LP	Female	Cryptorchidism, bilateral vesicoureteral reflux, ureterohydronephrosis, gonadectomy (fibrosis, structures similar to ducts, fragments of the uterine tube with sclerosis)	(13)
7	2022^d^	(1) Abnormal external genitalia with 46,XY (2) The mother of the child was healthy during pregnancy (no infections, radiation, poisons, or drugs). (3) Previous results of whole-exome sequencing were negative. (4) AMH and INHB levels were below the reference range. Peritoneal exploration showed testicular atrophy or poor gonadal development. Ultrasonography showed no evidence of a uterus	40%	40% (2/5)	c.G923A, p.R308Q	P	Male	Cryptorchidism, micropenis	(6)
							Female	Clitoral hypertrophy	
8	2022	Patients with posterior hypospadias and 46,XY	47.62%	2.38% (1/42)	c.G923A, p.R308Q	P	Male	Hypospadias (scrotal), penis dysplasia, bilateral cryptorchidism	(12)
9	2023^e^	(1) Patients had a 46,XY karyotype confirmed by high resolution G-banding (2) Patients with external genital malformation, including female external genitalia, clitoromegaly, ambiguous external genitalia, perineal hypospadias and micropenis	64.3%	5.71% (4/70)	c.A760G p.I254V	VUS	Male	Micropenis, urethral meatus: perineal, patent ductus arteriosus	(11)
					c.G923A, p.R308Q	P	Male	Micropenis, urethral meatus: perineal	
							Male	Micropenis, urethral meatus: penile, right gonad position: inguinal	
					c.G1516A p.D506N	VUS	Male	Micropenis, urethral meatus: glandular, gonad position: inguinal	
10	2023	Patients of primary amenorrhea with 46,XY DSD and no menarche by the age of 13 or later	80.00%	4.00% (1/25)	c.C1877T, p.S626L	LP	Female	Primary amenorrhea, secondary sex characters: Tanner stage: B1P3A2	(9)
11	2024	(1) 46,XY DSD with atypical external genitalia (2) Undetected or hypoplastic testes in the scrotal structures or near the groin on ultrasound or MRI (3) The regression of Müllerian ducts observed on ultrasound, MRI, or laparoscopy (4) Serum testosterone levels or serum anti-Müllerian hormone levels below the lower limit of the reference range for boys of the same age group.	81.82%	45.45% (5/11)	c.G923A, p.R308Q	P	Male	Micropenis, hypoplastic gonads in the inguinal canal,	(5)
							Male	Micropenis, hypoplastic gonads in the inguinal canal,	
							Male	Micropenis, hypoplastic gonads in the inguinal canal,	
							Female	External genitalia: female-type, hypoplastic gonads in the inguinal canal,	
							Male	Micropenis, hypoplastic gonads in the inguinal canal,	
					c.A1882C, p.T628P	LP	Female	External genitalia: female-type, streak gonads in the abdominal cavity	
12	2024	ESTAND- primary spermatogenic failure	12.28%	0.77% (4/521)	c.2177dup, p.T727Dfs*60	LP	Male	Oligozoospermia	(14)
					c.A2698G, p.N900D	LP	Male	Non-obstructive azoospermia, sertoli cell-only syndrome	
					c.A2762G p.Q921R	LP	Male	Non-obstructive azoospermia, sertoli cell-only syndrome	
					c.G1156A p.G386S	LP	Male	Oligozoospermia, testicular microlithiasis, mild cognitive, memory and speech impairment	

Exclusion criteria: (a) 17ß-hydroxysteroid dehydrogenase 3 deficiency, 5α-reductase deficiency type 2, and androgen insensitivity syndrome; (b) Patients with dysmorphic features, developmental delay, and/or intellectual disability, as well as those displaying > 2 malformations besides the genital abnormalities; (c) Congenital adrenal hyperplasia (CAH); (d) Abnormalities such as dwarfism, psychomotor developmental abnormalities; (e) Patients with 17α-hydroxylase/17, 20-lyase deficiency (17-OHD). LP, likely pathogenic; P, pathogenic; GD, gonadal dysgenesis; ETRS, embryonic regression testicular syndrome; DSDUE, DSD of clinically unknown etiology; ACMG, American College of Medical Genetics and Genomics; AMH, anti-Müllerian hormone; VUS, variant of uncertain significance; ESTAND: the ESTonian ANDrology (ESTAND) cohort.

## 3 Non-impact of *DHX37* on 46,XX females

**Table 2** summarizes the profiles of female carriers. None had any clinical manifestations of DSD, and they were capable of normal reproduction. Thirteen families had 15 female individuals carrying the “P” or “LP” variant of the *DHX37* gene who were not affected and could have a normal pregnancy. However, male offspring who inherited the *DHX37* gene variant exhibited the DSD phenotype, And the mutation sites include p.R308Q, p.L467V, p.R674W, p.R671T, p.G478R, p.R627F, and p.S595F (2, 8, 15–18). This suggests that the *DHX37* mutation does not affect gonadal development in females with 46,XX. Therefore, this gene mutation should be mentioned during genetic counseling to avoid passing the mutation to offspring, which will lead to gonadal dysgenesis, burdening the family and child.

**Table 2:** Status of female carriers of *DHX37* gene mutations.

Case no.	Variant of *DHX37*	ACMG	Relationship with the proband	Asymptomatic carrier	Reference
1	c.G923A, p.R308Q	P	Mother	Yes	(5)
2	c.C1399G, p.L467V	VUS	Mother	Yes	(15)
3	c.C2020T, p.R674W	LP	Mother, grandmother	Yes	(16)
4	c.G2012C, p.R671T	P	Mother	Yes	(17)
5	c.G1432A, p.G478R	VUS	Mother	Yes	(18)
6	c.C1879A, p.L627F	VUS	Mother	Yes	
7	c.G923A, p.R308Q	P	Mother	Yes	(8)
8	c.C1430T, p.T477M	LP	Mother	Yes	
9	c.G923A, p.R308Q	P	Mother	Yes	(2)
10	c.C2020T, p.R674W	LP	Mother	Yes	
11	c.C2020T, p.R674W	LP	Mother, grandmother	Yes	
12	c.C1784T, p.S595F	LP	Mother, grandmother	Yes	
13	c.C2020T, p.R674W	LP	Mother	Yes	

There was no significant difference in the expression of *DHX37* between XX and XY individuals in the sex-determining embryonic gonads of mice (4). In human fetal testicular tissue, *DHX37* is detected in Sertoli cells and some spermatogonia, but not in germ cells (2). The *DHX37* gene may only function after sex differentiation, following the *SRY* gene, and simultaneously with *SOX8* or *SOX9* (19), which may be the main reason why it only affects gonadal development in 46,XY individuals but exerts no effect on 46,XX individuals.

## 4 Incomplete penetrance in 46,XY males

Several cases of male carriers (the father of the proband) with no evident phenotype or with normal fertility have been reported. In a Brazilian family, there were two male siblings with 46,XY DSD (ETRS, micropenis, and non-palpable) caused by the p.R308Q mutation in *DHX37*. Their father also carried the p.R308Q mutation but exhibited no related phenotype and had three healthy children (2). A French patient with 46,XY DSD (TRS) had a p.T477M homozygous mutation in *DHX37*, inherited from both parents, the fertile father of this patient was noted to have unilateral testicular agenesis (8). In an Algerian patient with 46,XY DSD, the main clinical manifestations were micropenis, bilateral cryptorchidism, non-palpable gonads, and poorly developed, fused labia caused by the p.R487H mutation in *DHX37*, inherited from a phenotypically an asymptomatic father (8). *DHX37* mutations may also result in incomplete penetrance in males with 46,XY.

## 5 Molecular mechanism of *DHX37* gene mutation causing disease

The majority of members of the RNA helicase family have been reported to be associated with neurological diseases (20, 21), and DHX37 is currently the only helicase gene that is associated with both neurological disorders and DSD (2, 22), its molecular mechanisms have been reported very little. In pseudomales (female-to-male sex reversals) of the Chinese tongue sole (*Cynoglossus semilaevis*), the expression of *DHX37* and other Z chromosome-specific genes, which are important for spermatogenesis maintenance, is lower than that in normal males (23). In zebrafish, *DHX37* can interact with GlyR1,3,4a transcripts, and *DHX37* gene defects (dhx37^nig1^ mutation) can cause splicing defects in the transcription process of GlyR1,3,4a subunits. The defects can also reduce mRNA levels and regulate glycinergic synaptic transmission, leading to an abnormal motor response (24). UTP14A activates the ATPase activity of DHX37 by binding to its carboxyl-terminal domain via conserved regions, thereby enhancing the binding of DHX37 to RNA and promoting ribosome synthesis (25). Activated DHX37 can displace box C/D snoRNA U3 from pre-ribosomal particles to ensure correct and orderly folding of ribosomal subunits (26, 27). In hepatocellular carcinoma cells, *DHX37* is highly expressed and promotes proliferation and cancer progression by interacting with PLRG1 and activating the expression of CCND1 (28). In human CD8 T cells, DHX37 could be a regulator affecting NF-κB signaling, T cell activation, and cytotoxicity (29). Nuclear stress, transient activation of the Wnt pathway, and elevated P53 have also been reported in individuals with *DHX37* defects (30). Overall, the pathogenic molecular mechanisms of *DHX37* and 46,XY DSD are not fully understood. These mechanisms may be involved in ribosome synthesis, cell cycle regulation, and the NF-κB and Wnt pathways. It is currently unclear how this gene is integrated into the genetic network of differences in sexual development; therefore, further validation experiments are required.

## 6 Treatment of patients with 46,XY DSD associated with *DHX37* mutation

The treatment of 46,XY DSD related to *DHX37* deficiency is very difficult; there are currently no specific treatment methods available, with very limited reference cases (5, 31). We reviewed all current publications and only six cases with specific treatments have been reported, as summarized in **Table 3**. Surgical procedures, such as testicular fixation or orchidectomy, are one of the main coping strategies to avoid malignant changes in gonadal tissue; however, there has been no significant improvement in testicular function after surgery. Androgen therapy is also necessary and can help increase the penis size of patients to some extent, maintain normal adrenal function, and support pubertal development; however, it cannot improve fertility. Patients who present as female usually need to undergo external genital plastic surgery. Detailed prognostic and follow-up information is lacking, possibly because *DHX37* has only been associated with 46,XY DSD in the last few years, and documenting the dynamic development of infants, children, and adolescents is time-consuming and labor-intensive. Molecular mutations can lead to diseases, and only treatment at the genetic level can truly solve this problem. With advancements in science and technology, and the deepening of knowledge on genetic diseases, we look forward to a cure for genetic diseases in the future.

**Table 3:** Treatment and prognosis of patients with 46,XY associated with *DHX37* gene mutations.

Case no.	Gene mutation	ACMG	Clinical manifestations	Therapy	Prognosis	Reference
1	*DHX37*: c.G923A, p.R308Q	P	Micropenis and no palpable testes, the stretched penile length was 1.0 cm at the age of 11 months.	Hypoplastic gonads in the inguinal canal were removed via surgical procedures at 1 year and 4 months.	Unknown	(5)
2	*DHX37*: c.G923A, p.R308Q	P	Micropenis and no palpable testes, the stretched penile length was 1.2 cm at the age of 2 months.	Hypoplastic gonads in the inguinal canal were removed by surgery at 1 year and 9 months.	Unknown	
3	*DHX37*: c.G923A, p.R308Q	P	Raising gender as female with labial fusion and vulval closure	Partial urogenital mobilization and bilateral gonadectomy were performed at 1 year and 8 months	Unknown	
4	*DHX37*: c.G923A, p.R308Q	P	–	Urethroplasty was performed at 2 years and 4 months and orchiopexy at 2 years and 8 months.	The stretched penis length was 30 mm (-2.7 SD), the hypoplastic gonads were located in the scrotums each with a volume of 0.5 mL at 3 years and 9 months.	
5	*DHX37*: c.G923A, p.R308Q	P	Ambiguous external genitalia with a 1.0 cm phallus at one month of age, entered normal puberty at 12 years of age but presented with low testosterone levels at 17 years of age, high levels of FSH (12.40 IU/L), normal levels of LH (2.70 IU/L), and total testosterone (154.00 ng/dL)	Testosterone (1 ampoule IM every 15 days) at 17 years old.	At 21 years old: Tanner: G5P5, small testes of 6 mL each, high levels of FSH (34.50 IU/L) and LH (12.00 IU/L), azoospermia.	(31)
6	*DHX37*: c.C1399G, p.L467V NR5A1: c.288_304del, p.M98Glyfs*45	VUS; LP	Primary amenorrhea, spontaneous pubarche and no thelarche, female external genitalia with clitoromegaly (3 cm), no palpable gonads, and uterus absent	Gonadectomy, oral estrogenic replacement therapy with T5P5.	Normal adrenal evaluation.	

ACMG, American College of Medical Genetics and Genomics; LP, likely pathogenic; P, pathogenic; VUS, variant of uncertain significance.

## 7 Conclusion

In summary, sixty patients had DHX37-related 46,XY DSD, with a total of 25 variant sites. The p.R308Q and p.R674W variants were the two most common mutation hotspots, accounting for 36.67% and 11.67% of cases, respectively. The gonadal development and fertility of female (46,XX) carriers of *DHX37* mutations are not affected; however, incomplete penetrance may be observed in males (46,XY). In DSD cohorts, *DHX37* gene mutations have different detection frequencies (0.77%–45.45%), whereas in TRS and 46,XY GD cohorts, they have a high rate of detection. The molecular mechanism of *DHX37* pathogenesis, the specific pathways of action, and target molecules remain uncertain; however, ribosome synthesis, cell cycle regulation, and the NF-κB and Wnt pathways are suspected to be involved. Surgical intervention and appropriate timing of hormone replacement therapy are commonly used for DHX37-related 46,XY DSDs, although there are limited reported cases. The maintenance of male function and fertility after treatment remains unknown. Gene therapy for genetic diseases may provide new opportunities for the treatment of these diseases in the future.
